# Virtual Cold Chain Method with Comprehensive Evaluation to Reveal the Effects of Temperature Abuse on Blueberry Quality

**DOI:** 10.3390/foods13233731

**Published:** 2024-11-21

**Authors:** Min Fu, Hui Liu, Wenbo Yang, Qiang Zhang, Zhenzhen Lv, Muhammad Nawaz, Zhonggao Jiao, Jiechao Liu

**Affiliations:** 1 Zhengzhou Fruit Research Institute, Chinese Academy of Agricultural Sciences, Zhengzhou 450009, China; fumin22@163.com (M.F.); yangwenbo@caas.cn (W.Y.); zhangqiang02@caas.cn (Q.Z.); lvzhenzhen@caas.cn (Z.L.); mnawazafzal@outlook.com (M.N.); jiaozhonggao@caas.cn (Z.J.); liujiechao@caas.cn (J.L.); 2 Zhongyuan Research Center, Chinese Academy of Agricultural Sciences, Xinxiang 453000, China

**Keywords:** blueberry, cold chain, temperature, quality, comprehensive evaluation

## Abstract

Blueberry is one of the most perishable fruits, and the postharvest supply chain environment temperature has great effects on fruit quality. In order to determine the critical steps referring to non-optimal conditions and the key quality indexes in response to temperature along the cold chain, 15 time–temperature scenarios were conducted simultaneously for the storage of blueberry fruits and 17 quality attributes were determined. The results indicated that different cold chain steps under abusing temperatures significantly affected blueberry qualities. Based on the comprehensive evaluation analysis, storage in farm at 8 °C and a 10 h delay in precooling were found to be the critical steps that had strong impacts on the qualities of blueberries, affecting 33 and 31 total qualities during shelf life, respectively. Furthermore, seven effective indexes, including the sensory scores, weight loss, decay rate, content of titratable acid, malondialdehyde, respiratory rate and antioxidant activities, were confirmed to be the key quality attributes influenced by the cold chain temperature. It is necessary to circulate postharvest blueberry fruits under relatively isothermal and optimum temperatures throughout the cold chain to maintain the preferred quality, especially at the initial stage of the supply chain.

## 1. Introduction

Blueberries have become highly appreciated for their health-promoting (nutraceutical) value and sensory characteristics. However, they are highly perishable and susceptible to rapid spoilage and dehydration [1], resulting in a strong seasonal availability, a short shelf life, and inevitable subsequent economic losses [2]. It is reported that the shelf life of fresh blueberries ranges from 1 to 8 weeks, depending on the method of harvest, stage of fruit ripeness, storage and transport conditions [3]. Methods of extending the postharvest supply time and improving the storage quality of blueberries have become foci of attention [4].

At present, many studies focus on more efficient, green and environmentally friendly comprehensive preservation technologies. Low-temperature storage is currently the most common and convenient preservation method used in the blueberry industry. Nunes et al. studied changes in the storage quality of blueberries under different temperature conditions (0 °C, 5 °C, 10 °C, 15 °C and 20 °C) and found a negative correlation between blueberry quality and temperature [5]. The application of air cold plasma at atmospheric pressure during blueberry storage can prolong the shelf life and improve the quality of blueberries [6]. An edible coating made from konjac glucomannan has a good preservation effect in maintaining blueberry quality [7]. These studies focused on the impact of different postharvest technologies on the quality of blueberry fruits, generally during static storage.

However, blueberry fruits are subjected to a dynamic supply chain after harvest and environmental conditions significantly affect their quality. The importance of research on blueberry preservation technology in dynamic circulation has become increasingly prominent. Cold chain circulation is an effective way to maintain fruit storage and shelf life quality. Temperature is considered to be a core factor in a cold chain, as it plays an essential role in physiological metabolism, enzyme activity and other important functions of fresh food. A constant cryogenic temperature environment is vitally important for cold chain circulation. Unfortunately, cold chains are often exposed to temperature abuse caused by discontinuous supply processes and unstable refrigeration equipment [8]. According to the report of Liu [9], the losses at storage are about 10–15% for fruits and 5–10% at the distribution stage in China, which contributes most during the whole cycle. Poor temperature conditions along the cold chain trigger a large amount of food loss and waste and lead to a lack of safe production [10]. Non-optimal temperature in cold chains results in fruit quality deteriorations such as weight loss, texture softening and nutritional compound loss [11,12]. Rediers et al. [13] found that large temperature fluctuations significantly increased the growth of *Escherichia coli* and *Enterobacteriaceae* bacteria in fresh-cut endive. Strawberries kept under a steady temperature possessed better sensory and physicochemical qualities than the fruit exposed to a simulated supply chain with unsteady temperatures [14]. Kelly et al. revealed that using non-optimal temperatures in the critical steps of the strawberry supply chain reduces the fruit quality [11].

The construction of a fruit cold chain system from harvesting to consuming is essential to ensure the quality of fruits and promote the development of the fresh blueberry market [15]. Knowledge of key temperature-control steps in a cold chain can help to focus efforts to minimize quality deterioration. At present, very few studies have investigated the relationship between cold chain temperature conditions and the quality of blueberries. Ktenioudaki et al. [12] examined the impacts of temperature deviation at different cold chain steps on the physicochemical quality of blueberry fruits, but the influences on shelf life were not continually tracked and the key qualities responding to temperature abuse were not evaluated. In order to address this gap, this study set isothermal and non-isothermal storage temperature conditions to simulate different cold chains, examined the impacts on the physicochemical quality of blueberries during 7 day cold chain and 7 day shelf life and identified the most critical steps from field to consumer. Furthermore, the core quality indexes that sensitively responded to temperature change and used to evaluate the comprehensive quality were confirmed. This research aimed to guide the improvement of cold chain parameters and provide a scientific reference for reducing quality losses of blueberries in the postharvest supply chain.

## 2. Materials and Methods

### 2.1. Plant Material

“Bluecrop” blueberries were supplied by a local blueberry farm (latitude 112°59′ N, longitude 33°36′ E and altitude 152 m) located in Lushan County in Pingdingshan City of Henan Province. The climate type is a warm temperate continental monsoon climate, with an average annual temperature of 14.8 °C and a mean annual precipitation pattern of 1000 mm. The blueberry trees were 7 years old and were grown in acid soil under conventional management in an open field, and the fruits were harvested by hand on 10th June of 2023 and then transferred to the laboratory within 3 h. Blueberries were selected for uniformity of color and size and without visible quality defects.

### 2.2. Simulated Cold Chain Design

**Figure 1:**
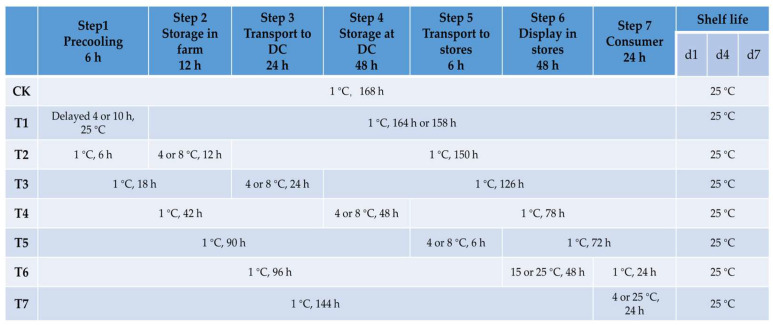
Time–temperature scenarios of simulated cold chain steps of blueberry fruits. CK: control group at isothermal temperature. T1–T7: different cold chains at non-isothermal temperatures. DC: distribution center. d1, d4 and d7: the 1st, 4th and 7th days of shelf life.

Typical cold chain steps used for fruit circulation are shown in Figure 1. The blueberry fruits were divided into 15 groups according to a completely random grouping method, which included 1 control condition (isothermal, 1 °C and 90% RH) and 14 different temperature cold chain conditions (non-isothermal, 90% RH), with reference to Kelly et al. [11] and Ktenioudaki et al. [12] with some modifications. Each group comprised 3 replicates with 2 kg blueberries in each replicate. A sample of 500 g blueberries was randomly selected from each replicate for decay rate and weight loss measurement. As shown in Figure 1, for each cold chain step simulated, only one step differed from the control. The blueberries were still kept at constant optimum conditions in other steps. The optimum condition for blueberry storage was set at 1 °C as per previous research [12]. According to the National Standards GB/T 22918-2008 [16] of China (Technical requirements for temperature-controlled transportation of perishable food) and GB/T 24616-2019 [17] (Packaging, labeling, transport and storage for chilled and frozen foods in logistics), the non-optimal temperature was set at 4 °C and a higher temperature of 8 °C was used as a comparation. The 12 h storage time on the farm was used to simulate a situation where the fruits are not delivered on the harvest day. The time (24 h) taken to transport the fruit from the farm to the distribution center (DC) was based on the distance from the blueberry-growing area in Henan to most of the distribution centers of other provinces. The temperatures of store display and consumer storage were set according to realistic scenarios of a sales display cabinet and household storage. After the virtual cold chain duration (d1) was complete, the qualities of the blueberries were investigated and the fruits were transferred to 25 °C conditions to continuously simulate 7 day shelf life.

### 2.3. Quality Evaluation

#### 2.3.1. Commercial Attributes

Firmness: A texture analyzer (TA-XT plus, Stable Micro Systems Ltd., Godalming, UK) was used for fruit flesh texture measurement. A sample of 30 blueberries was randomly selected from each group and the measurements were taken on the opposite sides of the blueberries in the equatorial region. A 2 mm diameter stainless-steel probe (P/2) was used with a test speed of 2 mm/s and penetration depth of 7 mm. The firmness of blueberry flesh was expressed as N (mean F 3:4).

Sensory evaluation: A total of 8 assessors were trained for the blueberry fruit sensory evaluation. Twenty blueberries per replicate were placed on transparent trays blindly labeled 1–8. Then, they were randomly given to the panelists. The judges of the panel individually evaluated the appearance, flavor, texture and taste of the fruits and gave scores from 1 to 9 for each characteristic. “Excellent” was represented by a score of 9, “good” by 7, “average” by 5, “poor” by 3 and “very bad” by 1. The panelists wrote their scores of each characteristic on paper and the average value was used for data analysis.

Decay rate and weight loss: Fruits that showed symptoms of fungal development, leakage or collapse were deemed to be decayed [18]. Weight loss was measured by weighing blueberry samples before storage and on the day of analysis.

#### 2.3.2. Biochemical Attributes

Content of total phenolics and anthocyanins: A total of 1.0 g of the blueberry homogenate was extracted by 10 mL ethanol via an ultrasonic bath for 10 min. The extract solution was centrifuged and the residue was extracted again. The supernatant was merged and used for the measurement of total phenolics, anthocyanins and antioxidant activity. The Folin–Ciocalteu method was used to determine the content of total phenolics as per Zhang et al. with some modifications [19]. The results were expressed as mg gallic acid equivalent (GAE) per 100 g of fresh fruits. Total anthocyanin was determined by the pH differential method [20]. The results were expressed as mg cyaniding-3-O-glucoside equivalent per 100 g fresh weight.

Content of ascorbic acid: The content of ascorbic acid was determined by molybdenum blue colorimetry. A total of 1.0 g of blueberry homogenate was weighed and 5 mL of oxalic acid–EDTA was added. The mixture was centrifuged for 10 min. Next, 1 mL of the supernatant was mixed with 0.1 mL of phosphoric acid–acetic acid solution and 0.2 mL of 5% sulfuric acid. Then, the mixture was shaken evenly and 0.4 mL of ammonium molybdate solution was added. The final volume of 5 mL was fixed with distilled water and the absorbance at 705 nm was measured after 15 min.

Content of soluble solids, soluble sugars and titratable acidity: Soluble solid content (SSC) measurement used a hand-held refractometer (PAL-1, ATAGO Co., Ltd., Tokyo, Japan). The titratable acidity was determined by titration with 0.01 mol/L NaOH and expressed as citric acid equivalent (%). The extraction of soluble sugar was performed as described by Zhang et al. with some modifications [21]. Soluble sugars were determined by HPLC (e2695, Waters Corp., Wilford, MA, USA), with a Waters Sugar-Pack I column (6.5 × 300 mm) and a 2414 refractive index detector (Waters, Wilford, MA, USA). The column temperature was set at 80 °C and 50 mg/L EDTA-2Na-Ca solution served as the mobile phase with a flow rate of 0.5 mL/min. The total content of sugars was calculated as the sum of the individual sugar contents.

#### 2.3.3. Physiological Attributes

Malondialdehyde (MDA) content: The content of MDA was determined as described by Ge et al. [22] with some modifications. A total of 0.5 g of the blueberry homogenate was added into 2.5 mL 100 g/L trichloroacetic acid (TCA). The mixture was centrifuged at 9755× *g* for 30 min at 4 °C. Then, 0.4 mL supernatant was mixed with 3 mL 6.7 g/L thiobarbituric acid (TBA) and incubated in a boiling water bath for 20 min. After cooling to room temperature, the absorbance at wavelengths of 450 nm, 532 nm and 600 nm was determined.

Respiration Rate: Respiration rate was measured by a Fruit and Vegetable Respirometer (SY-1022, Shijiazhuang Shiya Technology Co., Shijiazhuang, China). About 50 g of blueberry fruits were put in an air-sealed container which was connected to the respirometer instrument and measured for 1 h. The results were calculated based on the amount of CO_2_ accumulated per kg of fresh blueberry samples per h.

#### 2.3.4. Antioxidant Activity and Enzyme Activity Attributes

Antioxidant activities: Antioxidant activity was determined by DPPH· and ABTS·+ scavenging activity and ferric reducing antioxidant power (FRAP) assay, following the methods of Hu et al. [23], Huang et al. [24] and Pellegrini et al. [25].

Enzyme Activities: The peroxidase (POD) and polyphenolic oxidase (PPO) activities were determined as described by Manda-Hakki et al. [26] with some modifications. The POD reaction mixture included 3.0 mL 25 mmol/L guaiacol, 0.5 mL enzyme extract and 200 μL 0.5 mol/L H_2_O_2_. Using distilled water as the blank reference and H_2_O_2_ addition time as the starting time, the absorbance at 470 nm was recorded per 10 s and continuously for 3 min. The absorbance changes of 0.01 per min represented one enzyme activity unit (U). The PPO reaction system includes 2.9 mL 0.1 mol/L of catechol and 0.1 mL of enzyme supernatant. We measured the absorbance at 420 nm within 3 min reaction time. The absorbance changes of 0.01 per min represented one enzyme activity unit (U).

### 2.4. Statistical Analysis

All experimental data were analyzed using SPSS software (IBM SPSS Statistics 26). Analysis of variance (ANOVA) in conjunction with Duncan’s multiple-range test was applied to identify statistically significant differences compared to the control at *p* < 0.05. After normalization, principal component analysis (PCA) was used to select critical quality indexes with the dimensionality reduction factor. The score plot and cluster heatmap were performed by an online platform for data analysis and visualization on https://www.bioinformatics.com.cn (last accessed on 10 October 2024).

## 3. Results and Discussion

### 3.1. Impact of Simulated Cold Chain Steps on Blueberry Commercial Attributes

**Figure 2:**
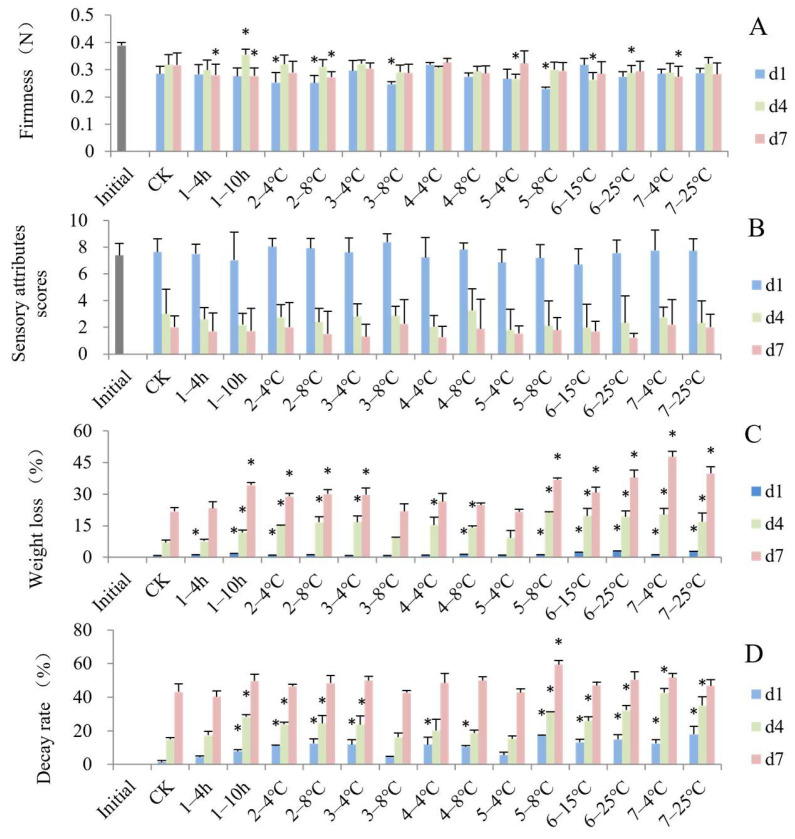
Impact of simulated cold chain steps on firmness (**A**), sensory scores (**B**), weight loss (**C**) and decay rate (**D**) of blueberries. * indicates significant differences (*p* < 0.05) between the temperature-increasing step and control group. Values are given as mean ± standard deviation (SD). CK: control group at 1 °C. 1—: delayed precooling for 4 or 10 h, 2—: stored in grower at 4 or 8 °C, 3—: transported to distribution center (DC) at 4 or 8 °C, 4—: stored at DC at 4 or 8 °C, 5—: transported to stores at 4 or 8 °C, 6—: displayed in stores at 15 or 25 °C, 7—: consumer storage at 4 or 25 °C. d1, d4 and d7: the 1st, 4th and 7th days of shelf life.

Fruit firmness is a crucial parameter that reflects senescence progress. As shown in Figure 2A, after the cold chain (d1), blueberry fruit firmness decreased compared to the initial fresh samples. The decline of fruit firmness was associated with increased water-soluble pectin content and decreased hemicellulose and cellulose [27]. However, the firmness of blueberries did not significantly decrease from d1 to d7. Similar results were reported along the blueberry supply chain by Ktenioudaki et al. [12]. This result may be related to the corrugation and thickening of the cell walls of epidermal and parenchymal cells caused by moisture loss in the outer cell layers [28]. At the end of the cold chains (d1), the firmness of the groups that were stored in the grower at 4 °C or 8 °C, transported to the DC at 8 °C and transported to stores at 8 °C significantly decreased compared to the control group. The fruits that underwent the steps of transportation to stores at 4 °C, displaying in store on d4 and delayed precooling, storage in the grower at 8 °C and consumer storage at 4 °C on d7 showed a significantly softer texture than the constant temperature group, respectively, indicating that the increase in temperature during the circulation process accelerated the softening. The same results were reported for the temperature abuse of peach [29], strawberry [11], sweet cherry [30] and cucumber [31]. The degradation of cell wall components and interconversion of protopectin were significantly accelerated in the isothermal treatment [26,27].

As shown in Figure 2B, the sensory scores of the d1 blueberries showed no significant difference from the initial fruits, but the scores declined significantly from d1 to d7, regardless of the time–temperature condition of each step. The fruit appearance in terms of shrinkage and decay showed the most significant changes and led to a rapid decline in sensory scores. There was no significant difference in sensory attribute scores for all groups exposed to the simulated supply conditions compared to the control. The results showed that the control group blueberries looked fresh with a firm texture and a lower decay incidence on d1; thereby, higher visual appearance and texture scores were obtained, but with a sour taste and light flavor. On the other hand, the non-isothermal groups exhibited poor overall appearance due to shrinkage and some disease incidence occurring. However, for these groups, the ripening and acid–sugar balance made the taste more preferable and highlighted the blueberry-like flavor. The sensory scores were the average of these characters and showed no significant differences among different groups. Bilbao-Sainz et al. [32] and Luo et al. [33] also reported that differences in the blueberry sensory scores of appearance, smell and taste were not statistically significant among different samples after 7 or 14 days’ storage.

The results showed that the weight loss of blueberries increased to 0.80~2.98% at the end of the cold chain (d1, Figure 2C). As the shelf life increased, the weight loss increased quickly to 21.55~47.84%. The weight loss of fresh produce is a result of the transpiration and respiration process. Compared to the control, most blueberries exposed to the temperature-fluctuating steps lost significantly more weight at the end of the cold chain (d1). In particular, blueberries stored at higher temperatures above 15 °C lost more moisture. The fruit weight loss while being displayed in store at 15 °C or 25 °C and while in consumer storage at 25 °C increased to 2.26%, 2.98% and 2.62% on d1, respectively. Additionally, the weight loss when precooling was delayed for 4 h or 10 h, or when the fruit was stored in the grower at 4 °C or in the DC at 8 °C, or transported to stores at 8 °C was significantly higher than the control group, indicating that delayed cooling and higher temperature significantly impacted the weight loss. Non-isothermal storage and transport enhanced the metabolic processes, increased the respiration and nutrient consumption as substrates, and induced weight loss and shriveling [31]. Delayed precooling resulting in severe mango weight loss has also been reported by Lee et al. [34]. Precooling is the first and one of the most important steps for the preservation of postharvest fruits. A lack of appropriate precooling will cause greater waste [35].

Similarly, a significant increase in the decay rate was also observed in all groups after the cold chain (Figure 2D). Most groups had significantly higher decay rates compared to the control on d1 and d4. The decay rate when fruits were transported to stores at 8 °C, displayed in stores at 25 °C or placed in consumer storage at 25 °C were higher than 14% on d1. These results agreed with previous research by Cao et al. [36], implying that the transportation temperature has a significant impact on the decay rate of citrus fruits. The decay rate of citrus transported at 15 °C was significantly higher than that at 10 °C and 5 °C.

### 3.2. Impact of Simulated Cold Chain Steps on Blueberry Biochemical Attributes

**Figure 3:**
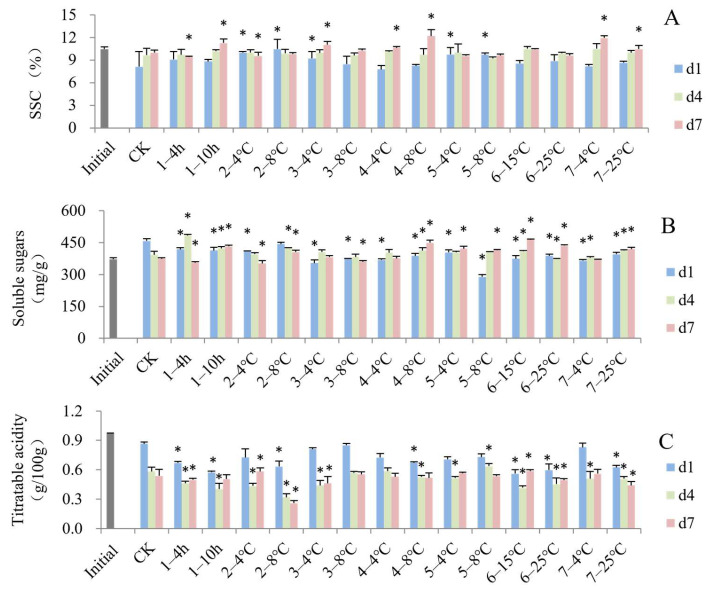
Impact of simulated cold chain steps on SSC (**A**), content of soluble sugars (**B**) and titratable acidity (**C**) of blueberries. * indicates significant differences (*p* < 0.05) between the temperature-increasing step and control group. Values are given as mean ± standard deviation (SD). CK: control group at 1 °C. 1—: delayed precooling for 4 or 10 h, 2—: stored in grower at 4 or 8 °C, 3—: transported to distribution center (DC) at 4 or 8 °C, 4—: stored at DC at 4 or 8 °C, 5—: transported to stores at 4 or 8 °C, 6—: displayed in store at 15 or 25 °C, 7—: consumer storage at 4 or 25 °C. d1, d4 and d7: the 1st, 4th and 7th days of shelf life.

The SSC of blueberries decreased after the simulated cold chain compared to the initial fresh samples (Figure 3A). SSC increased slightly in several groups during shelf life, which may be a consequence of postharvest metabolic processes such as starch conversion to sugar [37]. However, the SSC of some groups did not change. Similar results have been found by Eum et al. [38] and Chiabrando and Giacalone [39] in blueberry fruits. The SSC of the groups that were stored in the grower at 4 °C and 8 °C, transported to the DC at 4 °C and transported to stores at 4 °C or 8 °C were significantly higher than that of the control on d1. At the end of shelf life (d7), the SSC values of the groups that experienced delayed precooling by 10 h, transportation to the DC at 4 °C, storage at the DC and consumer storage at 4 and 8 °C were significantly higher than the control group. This may be due to the increase in temperature during the cold chain promoting the accumulation of SSC in blueberries. Furthermore, the decrease in moisture would increase the dry matter content of blueberry fruits.

In this study, there was an upward trend in total soluble sugar content in some groups (Figure 3B), in agreement with Wang et al. [40], who reported that sucrose will irreversibly decompose into glucose and fructose and lead to an increase in total sugar content during shelf life. After the virtual cold chain (d1), the soluble sugar content values at cold chain steps and different temperature conditions were significantly lower than the control, except for the fruits stored in the grower at 8 °C. The results of Kelly et al. [11] and Ktenioudaki et al. [12] also indicated that the soluble sugar content in groups of fruits with a temperature deviation from 1 °C in the cold chain were significantly reduced. Nunes et al. [41] found that fluctuating temperature resulted in a decrease in glucose content compared to constant temperature. These results may be due to an increase in fruit respiration metabolism involving the consumption of simple sugars under higher temperatures [42].

As shown in Figure 3C, compared to the fresh fruits, all groups of blueberries showed a decrease in titratable acid content after the cold chain (d1), and this decreasing trend continued throughout the 7 days of shelf life. The decreased acidity could be related to organic acids as substrates for respiratory metabolism [43]. In addition, there was significantly lower titratable acidity for the samples exposed to delayed precooling, stored in the grower at 8 °C, stored at the DC at 8 °C, and displayed in the store and under consumer storage at 25 °C compared to the control at the end of the cold chain (d1). An increase in temperature could result in a decline of malic acid and citric acid content [44,45]. The TA content of strawberries [11] and peaches [29] exposed to simulated temperature-fluctuating conditions was significantly lower than that in the constant temperature condition.

**Figure 4:**
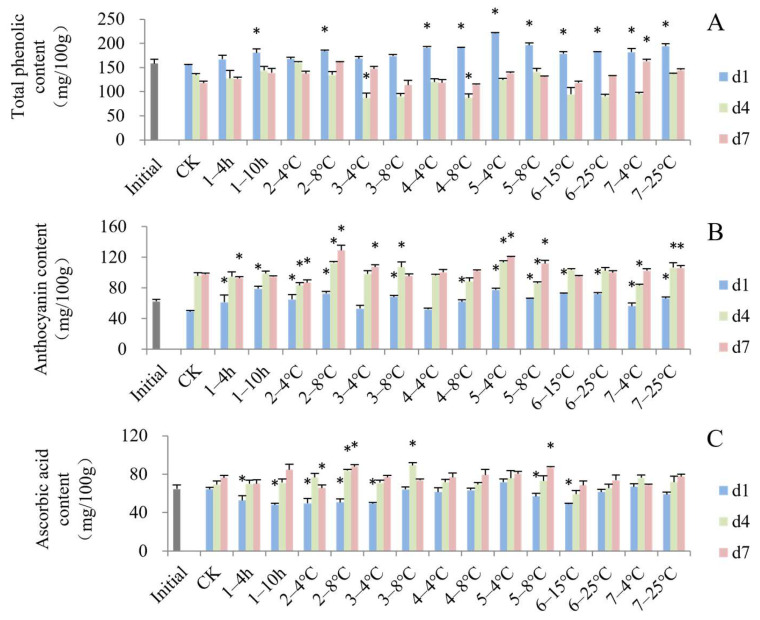
Impact of simulated cold chain steps on content of total phenolics (**A**), anthocyanins (**B**) and ascorbic acid (**C**) of blueberries. * indicates significant differences (*p* < 0.05) between temperature-increasing step and control group. Values are given as mean ± standard deviation (SD). CK: control group at 1 °C. 1—: delayed precooling for 4 or 10 h, 2—: stored in grower at 4 or 8 °C, 3—: transported to distribution center (DC) at 4 or 8 °C, 4—: stored at DC at 4 or 8 °C, 5—: transported to stores at 4 or 8 °C, 6—: displayed in store at 15 or 25 °C, 7—: consumer storage at 4 or 25 °C. d1, d4 and d7: the 1st, 4th and 7th days of shelf life.

Polyphenols, anthocyanins and ascorbic acid are important functional components with excellent contributions to antioxidant activity and stress resistance. In most cases on d1, blueberries in the different simulated cold chains showed higher contents of total phenolics (Figure 4A) and anthocyanins (Figure 4B) compared to the control. The increase in phenols and anthocyanins may be related to a stress response to adverse handling conditions [11]. In general, transport to stores at non-optimal temperature resulted in the highest content of total phenolics, and delayed precooling for 10 h resulted in the highest content of anthocyanins (Figure 4B) on d1. Some temperature-fluctuating steps led to significant lower content of polyphenols than the control, including transport to the DC at 4 °C and storage at the DC at 8 °C on d4 (Figure 4A). The cellular membranes collapsed and some polyphenol oxidase had the opportunity to catalyze the phenolic compounds as the shelf life increased [46]. The content of anthocyanins showed a different trend, generally increasing from d1 to d7. The groups that were subjected to storage in the grower at 8 °C, transportation to stores at 4 °C or 8 °C and consumer storage at 25 °C showed significantly higher anthocyanin content on both d4 and d7. This increasing trend is probably due to a higher concentration in fruits that have undergone water loss during their shelf life. Similar results had been previously reported in lowbush blueberries [47] and rabbit eye blueberries [48]. Nunes et al. [49] also reported that the anthocyanin content of strawberries exposed to a fluctuating temperature regime was higher than in a semi-constant regime.

Compared to the constant temperature condition and the initial fresh fruits, blueberries under higher-temperature cold chains had lower content of ascorbic acid on d1 (Figure 4C), indicating that some steps where the temperature rose accelerated ascorbic acid degradation, especially delayed precooling, storage in the grower, transport to the DC at 4 °C, transport to stores at 8 °C and displaying in stores at 15 °C. Cordenunsi et al. [50] assumed that low temperature has a protective effect on ascorbic acid content in fruits. The loss of ascorbic acid in fresh fruits and vegetables during storage has been attributed to cell wall damage and enzymatic oxidation [51]. From day 1 to day 7 of the shelf life, the content of ascorbic acid showed generally upward trends with anthocyanin content. The high level of water loss during shelf life tended to mask the loss of ascorbic acid, making it appear similar or even higher than the initial ascorbic acid value [52]. Moreover, the increase in ascorbic acid content also might be attributed to the synthesis of ascorbic acid from monosaccharides [53].

### 3.3. Impact of Simulated Cold Chain Steps on Blueberry Physiological Attributes

**Figure 5:**
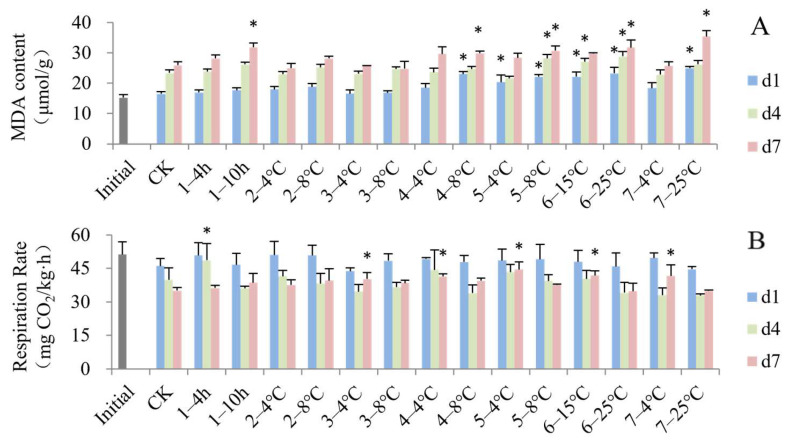
Impact of simulated cold chain steps on MDA content (**A**) and respiration rate (**B**) of blueberries. * indicates significant differences (*p* < 0.05) between temperature-increasing step and control group. Values are given as mean ± standard deviation (SD). CK: control group at 1 °C. 1—: delayed precooling for 4 or 10 h, 2—: stored in grower at 4 or 8 °C, 3—: transported to distribution center (DC) at 4 or 8 °C, 4—: stored at DC at 4 or 8 °C, 5—: transported to stores at 4 or 8 °C, 6—: displayed in store at 15 or 25 °C, 7—: consumer storage at 4 or 25 °C. d1, d4 and d7: the 1st, 4th and 7th day of shelf life.

Generally, the MDA content showed increasing trends during the shelf life as shown in Figure 5A. The MDA contents of the temperature-increasing groups at the latter-half stage (steps 4–7) were higher than the control group on d1, except for the group subjected to consumer storage at 4 °C. During the shelf life from d4 to d7, the MDA content of most simulated temperature-increasing steps were still significantly higher than the control group. MDA is the end product formed by the decomposition of certain primary and secondary lipid peroxidation products [54]. The concentration of MDA could reflect the degree of lipid peroxidation, membrane injury and fruit senescence, and an increase in MDA has been related to electrolyte leakage [22]. The non-isothermal environment accelerated the degree of membrane lipid peroxidation and cell membrane damage. Precooling treatment can prevent electrolyte leakage and an increase in MDA [55]. The MDA of peach fruits stored at room temperature or in non-isothermal conditions was accumulated much more than in those kept at constant and cold temperatures [29,56].

After the cold chain (d1), the respiration rate of all groups did not change significantly compared to the initial fresh samples (Figure 5B). This result might be due to the cold chain mainly being at a relatively cold temperature and inhibiting respiration. However, when the blueberry fruits were transferred to the shelf at room temperature, some groups experiencing temperature increases showed a higher respiratory rate compared to the constant temperature group, including those that experienced delayed precooling for 4 h on d4, transport to the DC at 4 °C, storage at the DC at 4 °C, transport to stores at 4 °C, displaying in store at 15 °C and consumer storage at 4 °C on d7. High temperature commonly enhanced the respiratory rate and promoted the appearance of respiratory peaks [56,57]. It was also confirmed that non-isothermal cold chain circulation significantly accelerated the postharvest respiration rate of peach fruits [29].

### 3.4. Impact of Simulated Cold Chain Steps on Blueberry Antioxidant Activity and Enzyme Activity

**Figure 6:**
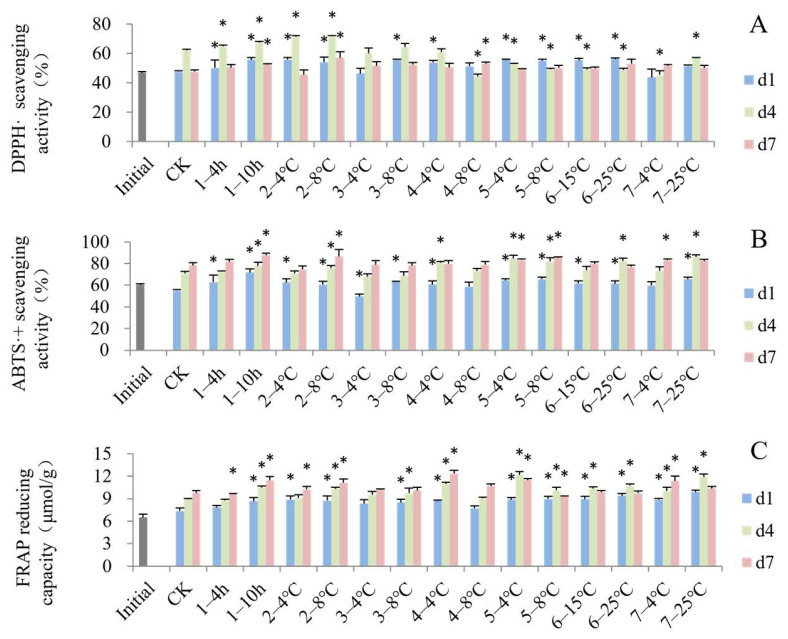
Impact of simulated cold chain steps on DPPH· scavenging activity (**A**), ABTS·+ scavenging activity (**B**) and FRAP reducing capacity (**C**) of blueberries. * indicates significant differences (*p* < 0.05) between temperature-increasing step and control group. Values are given as mean ± standard deviation (SD). CK: control group at 1 °C. 1—: delayed precooling for 4 or 10 h, 2—: stored in grower at 4 or 8 °C, 3—: transported to distribution center (DC) at 4 or 8 °C, 4—: stored at DC at 4 or 8 °C, 5—: transported to stores at 4 or 8 °C, 6—: displayed in store at 15 or 25 °C, 7—: consumer storage at 4 or 25 °C. d1, d4 and d7: the 1st, 4th and 7th days of shelf life.

Compared to the initial value of the fruit antioxidant activities at harvest, there was a significant improvement for most groups after the virtual cold chain (Figure 6). But the changes of three different antioxidant methods showed different trends. The DPPH· scavenging activity (Figure 6A) of some groups first increased and then decreased, but the ABTS·+ scavenging activity (Figure 6B) and FRAP (Figure 6C) showed a continuously increasing trend. The trends of ABTS·+ scavenging activity and FRAP were partially consistent with the changes in ascorbic acid content and anthocyanin content. Ascorbic acid and anthocyanins are important antioxidant compounds that influence the antioxidant activity of fruits. As reported by Lin et al. [58], a high antioxidant capability was mostly attributed to anthocyanins. In addition, most groups of blueberries exposed to the fluctuating temperature cold chain steps showed higher antioxidant capacity than the control group, similar to the results for ascorbic acid and anthocyanin content.

**Figure 7:**
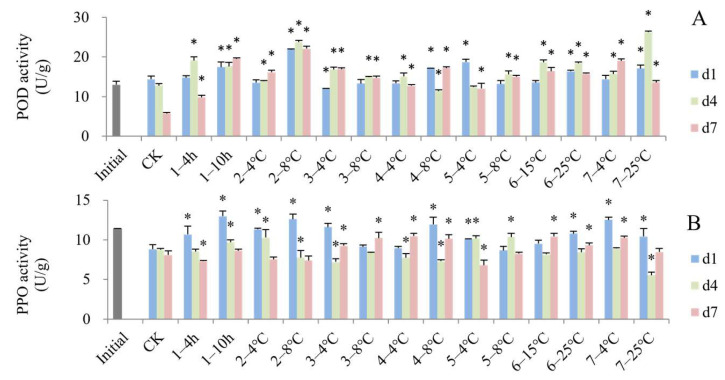
Impact of simulated cold chain steps on POD (**A**) and PPO (**B**) activity of blueberries. * indicates significant differences (*p* < 0.05) between temperature-increasing step and control group. Values are given as mean ± standard deviation (SD). CK: control group at 1 °C. 1—: delayed precooling for 4 or 10 h, 2—: stored in grower at 4 or 8 °C, 3—: transported to distribution center (DC) at 4 or 8 °C, 4—: stored at DC at 4 or 8 °C, 5—: transported to stores at 4 or 8 °C, 6—: displayed in store at 15 or 25 °C, 7—: consumer storage at 4 or 25 °C. d1, d4 and d7: the 1st, 4th and 7th days of shelf life.

As shown in Figure 7A, at the end of the simulated cold chain (d1), a total of six groups of blueberries showed a significant increase in POD enzyme activity compared to the control blueberries. As the shelf life increased, the POD activity of the control group decreased and some of the temperature-increasing groups possessed significantly higher POD activity on d4 or d7. The PPO activities of most groups with temperature increases were higher than the control at the end of the cold chain (d1), among which the groups that experienced delayed precooling for 10 h, storage in the grower at 8 °C and consumer storage at 4 °C showed the highest PPO activity (Figure 7B). Elevated temperature can sometimes lead to an increase in antioxidant enzyme activity. The POD and PPO activity of carambola fruits stored at 20 °C was significantly higher than those stored at 2 °C and 5 °C [59]. Wan et al. [60] revealed that hot air treatment of navel oranges enhanced the activity of POD enzymes.

### 3.5. Critical Qualities in Response to Cold Chain Temperature

In order to confirm the most influential indicators responding to temperature abuse along the cold chain, principal component analysis (PCA) was applied to describe all of the information contained in the dataset to detect the most important variables. Based on the eigenvalues > 1.0, three principal components were extracted (Table 1). According to Liu et al. [61], loading > 0.75 represents a strong effect of this variable on the principal component. The loading of sensory attributes (0.963) was the highest on PC1, followed by anthocyanins, ABTS·+, MDA, weight loss, decay rate, respiratory rate, TA, FRAP and ascorbic acid. The DPPH· scavenging activity possessed the highest loading for PC2. Among them, anthocyanins and ascorbic acid were closely related to the antioxidant activity, which could be summarized as a general antioxidant index including DPPH·, ABTS·+ scavenging activity and FRAP capacity. Therefore, the 17 quality indicators of blueberries could be simplified into 7 items, including sensory scores, MDA content, weight loss, decay rate, TA content, respiratory rate and antioxidant activity. These seven indicators are representative indicators that are sensitive to cold chain temperatures and could be selected as the critical evaluation qualities of blueberries.

### 3.6. Critical Temperature Control Steps Along Simulated Cold Chains

#### 3.6.1. Numbers of Qualities Affected by Different Simulated Cold Chain Steps

Table 2 shows the number of attributes that each step affected during shelf life. According to the maximum number of qualities impacted on d1, the top steps were delayed precooling for 10 h, storage in the grower at 8 °C, transportation to stores at 8 °C and displaying in stores at 25 °C, which all affected 12 quality attributes. On d4 of the shelf life, the steps that had the greatest impacts on blueberry qualities were storage in the grower at 8 °C, delayed precooling for 10 h, transportation to stores at 8 °C, displaying in stores and consumer storage at 25 °C. On d7 of the shelf life, the key step was storage in the grower at 8 °C. In summary, based on the sum number of the impacted quantities at d1, d4 and d7, the key cold chain steps were delayed precooling for 10 h, storage in the grower at 8 °C and transportation to stores at 8 °C, which affected 31, 33 and 31 qualities in total, respectively. A combination of higher temperature and longer duration time during the cold chain affected the fruit qualities. Most of the steps with the greatest number of impacting attributes were characterized by serious deviations (8, 25 °C) from optimum temperature or long-term duration (10 h). These results were partially similar to those found by Kelly et al. [11], with storage in a grower at non-optimal temperatures showing higher impacts on strawberry quality compared with other steps. Nunes et al. [62] revealed that delayed precooling resulted in strawberries showing less attractive physical and chemical qualities. Nowadays, great losses commonly occur in farms because of the dominant small-scale production farm system in China, which is inefficient in postharvest handling due to inadequate infrastructure and storage facilities, and the shortage of related preservation technology [35,63]. The lack of appropriate precooling has caused great postharvest wastage in China [35] and food waste at the consumer stage is also a large issue [63].

#### 3.6.2. Evaluation of the Critical Steps by Comprehensive Analysis

**Figure 8:**
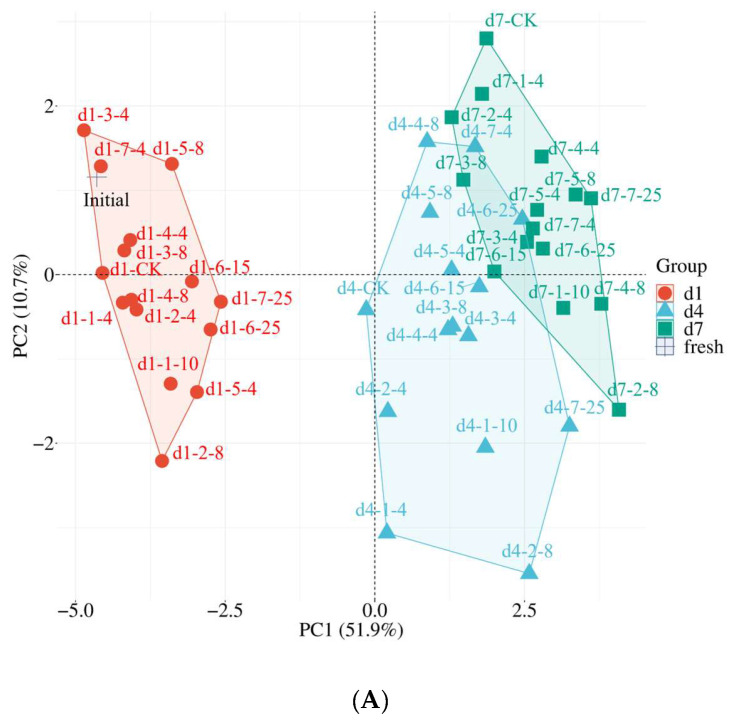

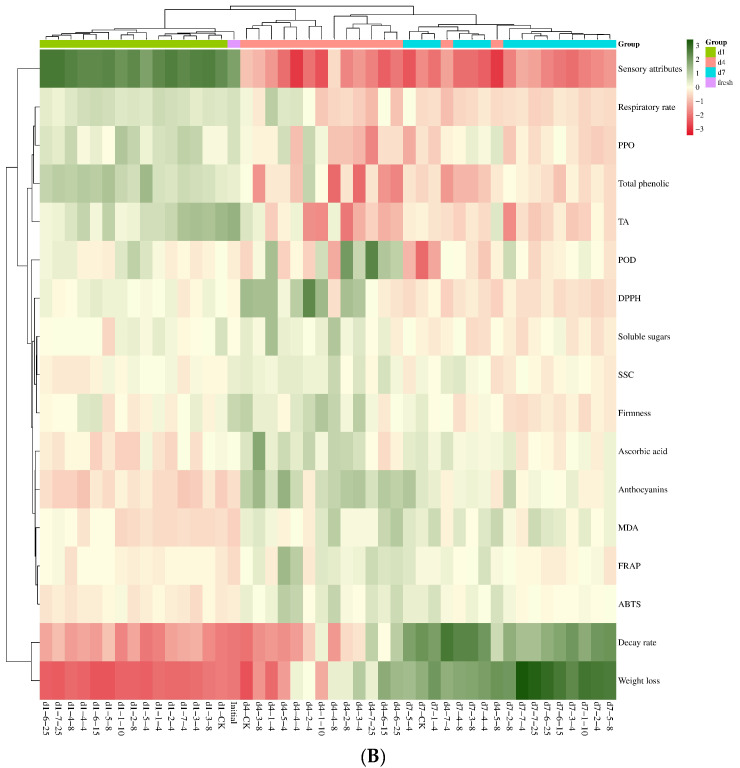
PCA score plot (**A**) and cluster heatmap (**B**) of blueberries under virtual cold chain steps. CK: control group at 1°C. 1—: delayed precooling for 4 or 10 h, 2—: stored in grower at 4 or 8 °C, 3—: transported to distribution center (DC) at 4 or 8 °C, 4—: stored at DC at 4 or 8 °C, 5—: transported to stores at 4 or 8 °C, 6—: displayed in store at 15 or 25 °C, 7—: consumer storage at 4 or 25 °C. d1, d4 and d7: the 1st, 4th and 7th days of shelf life.

A PCA score plot (Figure 8A) and cluster heatmap (Figure 8B) could help to visualize the evolution of blueberry fruits based on the comprehensive database. As shown in the PCA score plot, the blueberries at different shelf stages were situated in different quadrants. The groups of the initial fresh and d1 samples were located in the negative direction of PC1 and distinctly separated from the d4 and d7 samples. The samples of d4 and d7 were located far from the initial fresh sample, demonstrating notable distinctions from fresh samples. The distances of the groups stored at the farm at 8 °C, transported to the DC at 4 °C and transported to store were the farthest compared with the control on day 1. As for d4 and d7, the groups that experienced delayed precooling for 10 h, storage at the farm at 8 °C, storage at the DC at 8 °C and consumer storage showed the largest distances from the control group. The results were in agreement with Table 2, with the groups subjected to these steps significantly affecting the greatest numbers of qualities.

The cluster heatmap of the quality attributes and different cold chain steps is shown in Figure 8B. The initial fresh blueberry and d1 samples were clustered together, as with the score plot (Figure 8A). Blueberries that were transported to the store at 8 °C and in consumer storage at 4 °C on d4 were clustered with the d7 samples, which possessed lower sensory scores and higher weight loss and decay rates. On d1 of shelf life, most groups of blueberries suffering temperature abuse at a lower level (4 °C) were commonly clustered in the same group as the control and initial fresh samples, including the fruits that were subjected to delayed precooling for 4 h, stored at the farm at 4 °C, transported to the DC at 4 °C and in consumer storage at 4 °C, which showed equal sensory scores and TA content compared to the constant temperature group. However, the fruits subjected to delayed precooling for 10 h, farm storage at 8 °C and transportation to the store at 4 °C were clustered together, consistent with the PCA score plot. Similar trends were also found on day 4, with the cold chain steps with a lower temperature increase (4 °C) and a short delay time (4 h) clustered with the control group, which all showed higher content of anthocyanins, higher DPPH· scavenging activity and a lower decay rate. The groups of the last stage of cold chain steps (transportation to store, displaying in store and consumer storage), especially with temperature increases higher than 15 °C, usually clustered together, generally possessing the highest weight loss and decay rate and lower sensory and nutritional qualities. The results intuitively confirmed that these cold chain steps with rising temperature significantly accelerated quality loss in blueberries. Effective cold chain temperature management and the development of precooling, cold warehouses in farms and home refrigerator technology are significant to ensure fruit preservation quality and safety.

## 4. Conclusions

The quality properties of blueberry fruits were significantly influenced when exposed to some non-optimal temperature–time scenarios during simulated cold chains. Storage at the farm at 8 °C and delayed precooling by 10 h were confirmed to be the critical steps with the highest impacts on the quality attributes of blueberries. The temperature of farm cooling facilities needs to be maintained to not exceed 4 °C and the blueberries should be precooled quickly after harvest to maintain the preferred quality. Furthermore, sensory scores, weight loss, decay rate, TA content, MDA content, respiratory rate and antioxidant activity were considered to be the critical indexes for evaluating blueberry qualities and changed strongly in response to temperature abuse along the cold chain. By targeting these critical steps and the quality indexes, cold chain circulation temperature parameters could be controlled and optimized to reduce the waste and loss of blueberry fruits and would have a significantly positive advantage for promoting the development of the blueberry industry.

## Figures and Tables

**Table 1:** The eigenvalues, contribution rates and loadings of the principal components.

	PC1	PC2	PC3
Eigenvalues	8.820	1.811	1.447
Contribution rate (%)	51.882	10.654	8.510
Cumulative contribution rate (%)	51.882	62.535	71.045
Sensory attributes	0.963	0.057	0.113
Anthocyanins	−0.922	0.156	−0.025
ABTS·+	−0.916	−0.019	0.129
MDA	0.872	0.089	−0.162
Weight loss	0.868	0.222	−0.256
Decay rate	0.860	0.268	−0.262
Respiratory rate	0.816	0.181	0.228
TA	0.807	−0.336	0.015
FRAP	−0.798	0.017	0.220
Ascorbic acid	−0.765	0.030	−0.209
Total phenolics	0.690	0.179	0.356
SSC	−0.642	0.036	0.297
PPO	0.608	0.035	0.410
DPPH·	−0.124	0.784	−0.320
POD	−0.217	0.728	0.349
Soluble sugars	−0.233	0.573	0.044
Firmness	−0.191	0.013	−0.709

PC: Principal components.

**Table 2:** Number of qualities impacted by different simulated cold chain steps during shelf life.

Step	d1	d4	d7	Total
1—Delayed precooling for 4 h	8	5	8	21
1—Delayed precooling for 10 h	12	10	9	31
2—Stored in grower for 12 h at 4 °C	11	7	8	26
2—Stored in grower for 12 h at 8 °C	12	11	10	33
3—Transported to DC for 24 h, 4 °C	7	6	7	20
3—Transported to DC for 24 h, 8 °C	6	4	3	13
4—Stored at DC for 48 h, 4 °C	6	5	5	16
4—Stored at DC for 48 h, 8 °C	9	7	6	22
5—Transported to stores for 6 h, 4 °C	10	7	7	24
5—Transported to stores for 6 h, 8 °C	12	10	9	31
6—Displayed in stores for 48 h, 15 °C	11	9	6	26
6—Displayed in stores 48 h, 25 °C	12	10	6	28
7—Consumer storage for 24 h, 4 °C	7	8	9	24
7—Consumer storage for 24 h, 25 °C	11	10	7	28

DC: distribution center; d1, d4 and d7 mean the 1st, 4th and 7th days of shelf life.

## Data Availability

The original contributions presented in the study are included in the article, further inquiries can be directed to the corresponding author.
